# Fabrication and Characterization of Aluminum Nanoparticle-Reinforced Composites

**DOI:** 10.3390/polym12122772

**Published:** 2020-11-24

**Authors:** Seongbeom Jeong, Young Seok Song, Eunju Lim

**Affiliations:** 1 Department of Convergence Systems Engineering, Dankook University, Youngin 448-701, Korea; sungbum0414@naver.com (S.J.); ysong@dankook.ac.kr (Y.S.S.); 2 Department of Fiber System Engineering, Dankook University, Youngin 448-701, Korea; 3 Department of Science Education, Dankook University, Youngin 448-701, Korea

**Keywords:** acrylonitrile butadiene styrene, Al nanoparticle, composites

## Abstract

With the expanding use of polymers in modern our lives, there is an increasing need to manufacture advanced engineering polymeric parts in a systematic and inexpensive way. Herein, we developed an organic inorganic hybrid composite material with excellent mechanical properties by enhancing the dispersion and moldability of fillers. For this, we prepared and analyzed the physical properties of acrylonitrile butadiene styrene (ABS)/aluminum nanoparticle composites. Al nanoparticles of various sizes (20 nm and 40 nm) and concentrations (3, 6, 9, and 12 wt.%) were employed. The mechanical properties of the prepared composites were measured using a universal testing machine. Rheological and thermal analyses for the composites were carried out with use of a rheometer and a differential thermal calorimeter (DSC). We also conducted optical, chemical, electrical, and morphological property studies of the samples in order to help design and produce high-performance engineering products.

## 1. Introduction

A growing demand for high-performance and fine precision plastics has initiated the incorporation of composite materials in polymer processing technology [1,2,3]. In addition, it is critical to develop new engineering composites as hybrid organic inorganic materials rather than as single material. Currently, many engineering polymeric materials, such as polycarbonate (PC), nylon, polyester, urethane, and acrylonitrile butadiene styrene copolymer (ABS), are employed in a wide range of applications [4,5,6]. Moreover, the processing technologies for composite materials are being developed to produce structured products with excellent mechanical properties and geometrical precision. The physical properties of a product depend on the particle size, distribution, shape, molecular weight, melting point, and viscosity of the polymer used [7,8,9,10,11]. In the case of reinforcing materials used in composites, special properties such as high fluidity, thermal conductivity, and interfacial bonding strength, are required [12,13]. Efforts are ongoing for enhancing not only the functional properties of composites but also their aesthetic sensibility.

In this study, we fabricated metal nanoparticle-embedded composites via extrusion and injection molding. Aluminum nanoparticles of various sizes were incorporated into the composites and the effect of their loading was evaluated [14,15]. The development of conventional metal-like aesthetic materials was implemented by using micro-sized metal particles, since the glossiness is affected by the ratio of the content of micro-nanoparticles. The rheological properties of the metal composites, such as viscosity, storage modulus, and loss elastic modulus, were analyzed. We evaluated the metal-like properties of ABS/Al nanocomposites after injection molding. In order to improve the gloss and surface properties, the composite was irradiated with an ion beam, and the electrical properties of the composite were analyzed. We analyzed the glossiness, color, surface roughness, mechanical strength, etc., of the composites.

## 2. Materials and Methods

### 2.1. Materials Preparation

The organic material purchased to produce the organic/inorganic nanocomplex was acrylonitrile butadiene styrene (ABS) from LG Chem., Seoul, Korea. The used inorganic materials were aluminum nanoparticles, which have two particle sizes of 20 nm and 40 nm from CN vision. An extruder (Thermo Fisher Scientific’s Process 11 Parallel Twin-Screw Extruder, Waltham, MA, USA) was used to integrate Al nanoparticles into the ABS polymer. Prior to the extrusion, the material was stored in a dryer for 24 h. The 20 nm and 40 nm Al nanoparticles were dispersed in ABS at a weight ratio of 0, 3, 6, 9, and 12. The temperature and rotation speed applied to the extruder were 235 °C and 250 rpm, respectively. Resin preparation (Thermo Fisher Scientific’s Minijet Pro Piston Injection Molding System) was carried out for 5 s at 400 bar under conditions of cylinder 400 °C and mold 120 °C. Nitrogen and chromium ion beams were applied to increase the surface gloss of the ABS/Al nanocomposites. Ion beam irradiation was performed in the Korea Multipurpose Accelerator Complex (KOMAC, Seoul, Korea). The energy of the ion was 100 KeV, and the dose was 10^13^, 10^14^, and 10^15^ ions/cm^2^.

### 2.2. Methods

The rheological properties of the Al/ABS composites were measured using a rheometer (MCR302, Anton Parr, Graz, Austria) [16,17,18,19]. For rheological measurements, disk-shaped parts fabricated via injection molding were employed. The shear viscosity was measured in the shear rate range 0.01–1000 s^−1^. The oscillatory shear test was conducted in the frequency range 0.1–100 rad s^−1^. The storage modulus (G′) and loss modulus (G″) were analyzed [20]. Tensile tests were performed using a universal testing machine (UTM, Instron, Norwood, MA, USA). Young’s modulus, yield strength, and elongation at break were measured. To confirm the microstructure morphology of the nanocomposite, a scanning electron microscope (SEM, S-4700, Hitach, Tokyo, Japan) was used to observe the dispersion of nanoparticles in the sample. The thermal properties of the samples were measured using differential scanning calorimetry (DSC, Q2000 system, TA instrument, New Castle, DE, USA) [21,22,23,24,25,26]. Stopping and range of ions in matter (SRIM) simulation was performed to estimate the thickness of the ion beam penetration into the composite, and ion beam injection was performed at different flow rates/energies using two ion species (N and Cr) at room temperature. The beam flux was maintained at a fixed value. In order to evaluate the electrical features, Ag electrodes were fabricated at 1 cm intervals parallel to the sample. A voltage up to 5 V was applied to the sample at 0.1 V intervals. Current–voltage (I–V) observations were made using a semiconductor parameter analyzer (HP 4155A) [27,28].

## 3. Results and Discussion

**Figure 1:**
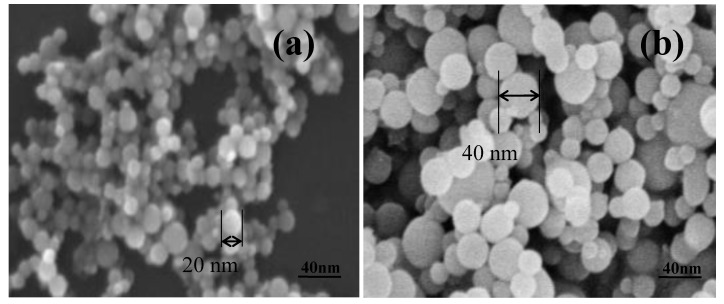
SEM images of Al: (**a**) 20 nm and (**b**) 40 nm nanoparticles.

**Figure 2:**
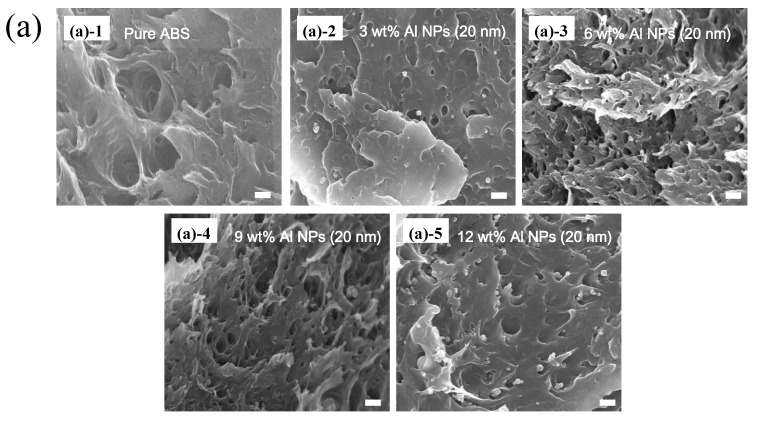

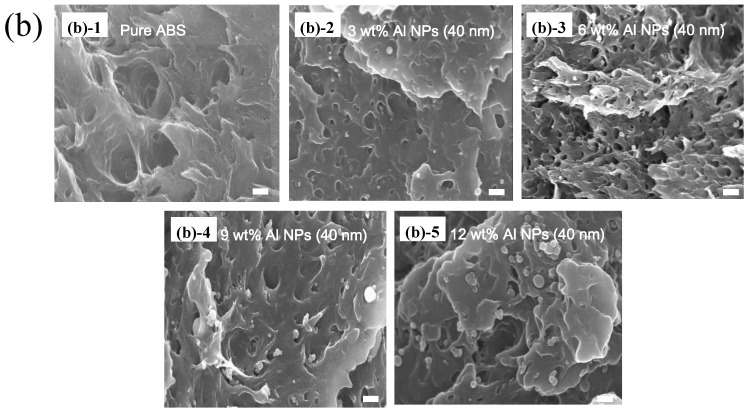
SEM image of cross-section of composite material of ABS and Al: (**a**) 20 nm and (**b**) 40 nm nanoparticles from 0 to 12 wt.%.

**Figure 3:**
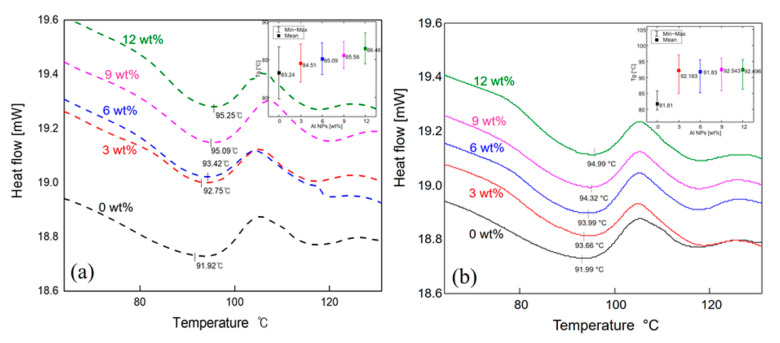
DSC of ABS/Al (**a**) 20 nm and (**b**) 40 nm nanoparticle composites.

Figure 1 shows the SEM images of the Al nanoparticles of sizes 20 nm and 40 nm. The spherical nanoparticles were dispersed in the ABS plastic with the use of an extruder. Figure 2a,b present the SEM images of the fractured surface. The nanoparticles were found to be evenly dispersed. In addition, as expected, more particles were observed as the particle loading increased. Figure 3 shows the DSC results of the composites. The effects of the particle content and the size of the nanoparticles on the glass transition temperature (T_g_) were analyzed. The transition temperatures of the 20 nm and 40 nm particle-embedded composites were found to increase with increasing the particle content from 0 to 12 wt.%. The change in the glass transition temperature is shown to be relatively large for the 40 nm particle-reinforced composite. In the case of the 40 nm particle-reinforced composite, the effect of particle addition was dramatic, but there was no significant difference between the samples with weight percentages of 3 to 12 %. This may be due to the decrease in the molecular mobility of ABS. 

**Figure 4:**
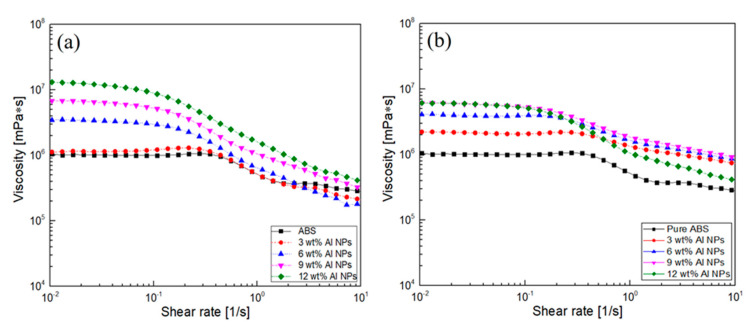
Viscosity of ABS/Al: (**a**) 20 nm and (**b**) 40 nm nanoparticle composites.

Rheology is a powerful tool for characterizing the internal structure of complex materials [16,17,18,19]. For instance, the viscoelastic characteristics can provide a variety of information regarding the internal structure of the test material such as particle dispersion, size, shape, and surface properties [29]. Figure 4a,b present the viscosity of the nanocomposites filled with 20 nm and 40 nm aluminum nanoparticles, respectively. The graph shows the so-called zero shear-rate viscosity and shear-thinning behavior. This means that the composite materials can be modeled with the help of the Cross-WLF model: η = η_∞ + (η_0 [−η]_∞)/(1 + (Cγ)^(1 − n)), where η_∞ is the infinite shear-rate viscosity, η_0 is the zero shear rate viscosity, C is a time constant, and n is the power law index. The model is a widely used rheological model for injection molding. As the filler loading increased, the resulting viscosity also increased. Interestingly, the nanocomposites filled with the 20 nm nanoparticles showed higher viscosity than those with the 40 nm ones. This is due to the fact that one had a higher effective volume of filler than the other. When a shear stress was applied to the samples, the flow resistance decreased. This is because the ABS molecules and Al nanoparticles were oriented in the direction of the shear flow. 

**Figure 5:**
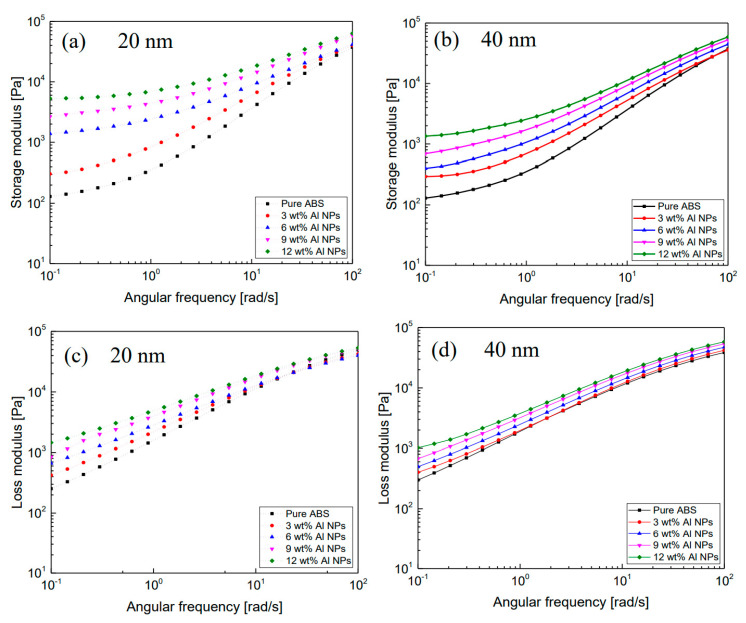
Storage modulus (G′) and loss modulus (G″) curves of ABS/Al 20 nm and 40 nm nanoparticle composites. (**a**,**c**) are ABS/Al 20 nm, (**b**,**d**) are the storage modulus and loss modulus curves of the 40 nm nanoparticle composite.

The small-amplitude oscillatory shear test (SAOS) was used for nondestructive analysis of viscoelastic materials [30]. Figure 5a,b show the storage modulus of the composites filled with 20 nm and 40 nm Al nanoparticles. The storage modulus increased with increasing filler loading. In particular, the composites with 20 nm particles showed higher modulus values than those with 40 nm particles, which indicated the so-called size effect, that is, larger particle matrix interaction. In addition, the composites reinforced with 20 nm particles exhibited more solid-like behavior in the low-frequency region than those with 40 nm particles. The loss modulus showed a similar behavior to the storage modulus as presented in Figure 5c,d. 

**Figure 6:**
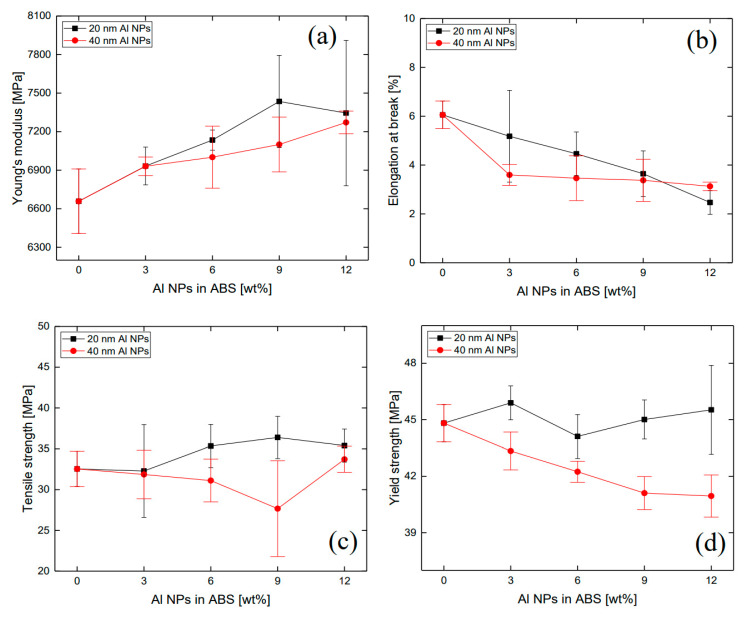
(**a**) Young’s modulus, (**b**) elongation at break, (**c**) tensile strength, and (**d**) yield strength results of ABS/Al composites.

The viscoelastic characteristics are correlated to the mechanical properties of the samples. Figure 6a shows the Young’s moduli of the composites. The elastic modulus increased as the Al particle concentration increased. Similar to the results of the storage and loss moduli, the 20 nm particle embedded composite was found to show higher elastic modulus than the 40 nm particle-filled composite. Figure 6b shows that the elongation at break had a tendency to gradually decrease as the concentration of aluminum nanoparticles increased. In the case of the 20 nm particle composite, the elongation at break decreased linearly. The elastic modulus and elongation at break showed an inverse relationship with each other. The tensile strength results are shown in Figure 6c. The tensile strength of the 20 nm particle composite had a tendency to increase as the concentration of particles increased, but that of the 40 nm particle composite showed a different trend. Figure 6d shows the yield strength of the composites. The yield strength indicates the degree of maximum stress that a material can undergo without causing plastic deformation. Plastic deformation is a state in which the material deforms permanently due to slip. For the composite filled with 40 nm particles, the yield strength decreased with increasing particle loading. 

**Figure 7:**
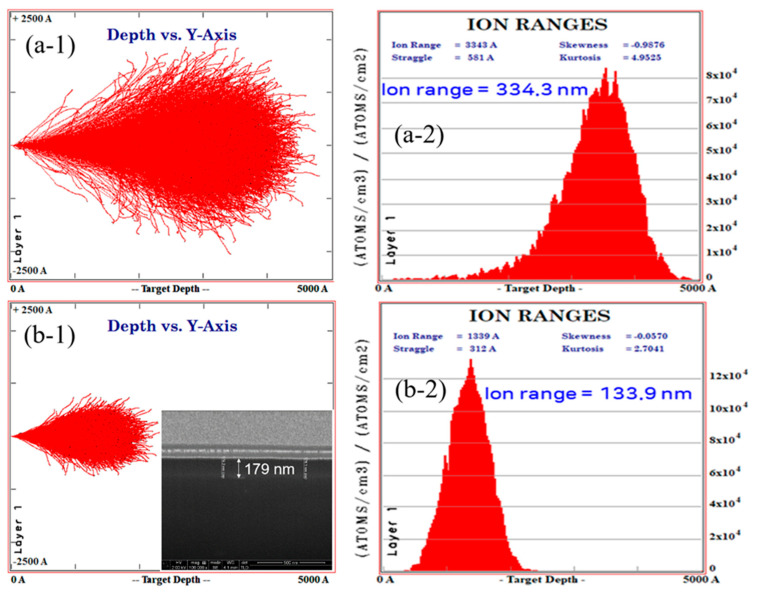
(**a-1**,**a-2**) Nitrogen and (**b-1**,**b-2**) chromium ion beams irradiation by SRIM simulation result. The inset figure in (**b-1**) shows the FIB image investigated from the side after irradiating chromium on the composite material.

**Figure 8:**
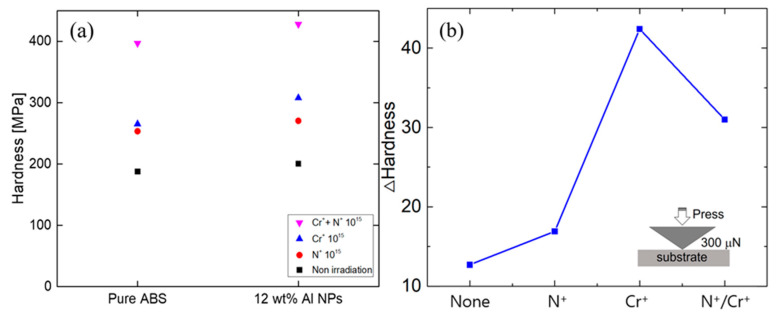
(**a**) Nanoindentation results of nitrogen and chromium ion beams irradiation composite samples and (**b**) difference value of nanoindentation results. (**b**) The inserted figure is a simplified structural image of the nanoindenter.

**Figure 9:**
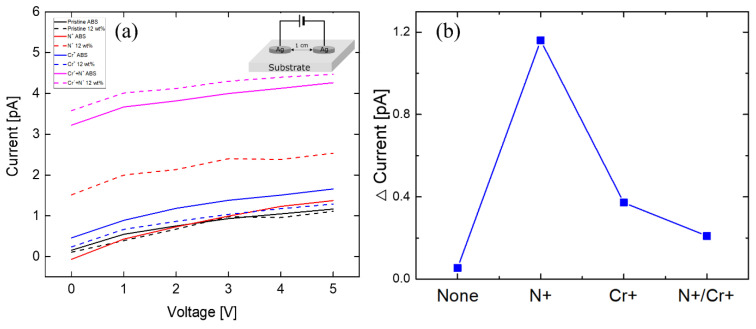
(**a**) Investigation of surface electrical properties through I–V measurement after nitrogen ion beam and chromium ion beam irradiation. (**b**) The difference in current when a voltage of 5 V was applied is shown. (**a**) Inset image shows I-V measuring element.

To enhance the surface properties, the composites were irradiated with a nitrogen gas ion beam and a chromium metal ion beam [31]. Figure 7 shows the penetration depth calculated through the SRIM simulation [32]. It was shown in the SRIM simulation results that the nitrogen ion beam penetrated into the surface by about 400 nm (Figure 7(a-1,a-2)), and the chromium ion beam penetrated into the surface by about 200 nm (Figure 7(b-1,b-2)). The inset in Figure 7(b-1) shows the FIB image irradiated from the side after chromium irradiation on the composite material. The difference between SRIM simulations and experiments indicates that not all beam irradiations lead to the contribution to pore formation onto the surface. When nitrogen and chromium ion beams were irradiated, small pores were formed on the surface due to ion size or a beam that had not yet penetrated. The corresponding experiments were divided into three cases: a nitrogen ion beam, chromium ion beam, and nitrogen and chromium. Figure 8a,b show the results of nanoindentation tests. The samples were irradiated with 100 eV ion beam energy at a dose of 10^15^ ions/cm^2^ in ABS and ABS/Al 12 wt.%. The ion beam effect was analyzed. Irradiating a composite surface with an ion beam increased the hardness of the surface depending on the type and mixing of the ion beam. In the case of chromium beam irradiation, the mechanical strength was the highest among the samples. This is because when the polymer material is irradiated with an ion beam, the surface hardness and density change, increasing the mechanical strength. Also, the surface structure of the penetration layer varies depending on the type of irradiation ion beam. To determine the surface conduction characteristics of the samples, the current voltage relation was analyzed after the Al metal electrode was positioned in parallel with the samples. Figure 9a compares the current values from 0 to 5 V when three types of ion beams were applied to the samples. In Figure 9b, the current difference at a voltage of 5 V was replotted and analyzed according to the type of ion beam irradiation. The difference in surface resistances in the presence and the absence of metal nanoparticles was the largest in the case of nitrogen ion beam irradiation, and the value of conductivity of the ion beam type was the highest in the case of chromium/nitrogen ion beam mixed irradiation. The ion beam irradiation can change macromolecular structures, e.g., the formation of new double bond structure or cross-linking structure. More specifically, when the nitrogen ion beam is imposed, the C–N and C–C bond can be formed, resulting in an increase in electrical conductivity [33]. 

**Figure 10:**
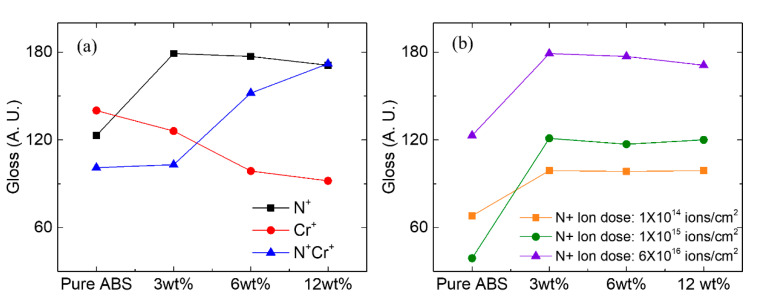
(**a**) Change of glossiness after irradiation with (**a**) nitrogen ion beam according to difference in irradiation amount and (**b**) nitrogen, chromium, nitrogen, and chromium ion beam irradiation under 1.8 × 10^16^ ions cm^−2^.

Figure 10 shows the result of the surface gloss of the composite. The effects of the ion beam types and ion beam doses were experimentally analyzed. Figure 10a shows the gloss results of the composite material. In the case of nitrogen ion beam irradiation, the gloss was greatly increased at a small amount (3 wt.%) of Al nanoparticles. There was no significant difference in gloss when Al metal nanoparticles at 6, 9, and12 wt.% were dispersed. In the case of chromium ion beam irradiation, the glossiness tended to decrease as the content of Al nanoparticles increased from 3 to 12 wt.%. In the nitrogen/chromium ion beam irradiation, the gloss gradually increased as the content of nanoparticles increased from 0 to 12 wt.%. The most effective condition for gloss is the case of nitrogen ion beam irradiation at a particle content of 3 wt.%. When the composite material containing Al nanoparticles was irradiated with a nitrogen ion beam, not only structural changes of C–C, C–H, and C–O bonds but also changes in the surface’s structure occurred. This led to changes in glossiness [33,34]. Figure 10b shows the change in gloss caused by the change in nitrogen ion beam dose. The gloss increased as the dose increased. Increased ion beam doses affected the surface density and cross-linking structure.

## 4. Conclusions

In this study, we fabricated organic inorganic composites by incorporating Al nanoparticles in an ABS polymer. The composites were extruded and injection-molded for experimental analyses. The mechanical, thermal, optical, chemical, and electrical properties of the composites were evaluated. The effects of nanoparticle size, concentration, and type of ion beam were analyzed. It was found that the composites prepared with 40 nm particles showed significantly enhanced thermal and mechanical properties. Also, the electrical properties of the polymer material could be improved by adjusting the nanoparticle content and ion beam irradiation. This study is expected to help to provide a way to design and manufacture metal nanoparticle embedded polymeric composites for high performance engineering products.
